# Domain-Specific Effects of Prenatal Exposure to PCBs, Mercury, and Lead on Infant Cognition: Results from the Environmental Contaminants and Child Development Study in Nunavik

**DOI:** 10.1289/ehp.1206323

**Published:** 2014-01-17

**Authors:** Olivier Boucher, Gina Muckle, Joseph L. Jacobson, R. Colin Carter, Melissa Kaplan-Estrin, Pierre Ayotte, Éric Dewailly, Sandra W. Jacobson

**Affiliations:** 1 Centre de Recherche du Centre hospitalier universitaire de Québec, Québec City, Québec, Canada; 2 Département de psychologie, Université de Montréal, Montréal, Québec, Canada; 3 École de psychologie, Université Laval, Québec City, Québec, Canada; 4 Department of Psychiatry and Behavioral Neurosciences, Wayne State University School of Medicine, Detroit, Michigan, USA; 5 Department of Pediatrics, Division of Emergency Medicine, Columbia University College of Physicians and Surgeons, New York, New York, USA; 6 Department of Psychology, Wayne State University, Detroit, Michigan, USA

## Abstract

Background: Polychlorinated biphenyls (PCBs), methylmercury (MeHg), and lead (Pb) are environmental contaminants known for their adverse effects on cognitive development.

Objectives: In this study we examined the effects of prenatal exposure to PCBs, MeHg, and Pb on cognitive development in a sample of Inuit infants from Arctic Québec.

Methods: Mothers were recruited at local prenatal clinics. PCBs, mercury (Hg), Pb, and two seafood nutrients—docosahexaenoic acid (DHA) and selenium (Se)—were measured in umbilical cord blood. Infants (*n* = 94) were assessed at 6.5 and 11 months of age on the Fagan Test of Infant Intelligence (FTII), A-not-B test, and Bayley Scales of Infant Development–2nd Edition (BSID-II).

Results: Multiple regression analyses revealed that higher prenatal PCB exposure was associated with decreased FTII novelty preference, indicating impaired visual recognition memory. Prenatal Hg was associated with poorer performance on A-not-B, which depends on working memory and is believed to be a precursor of executive function. Prenatal Pb was related to longer FTII fixation durations, indicating slower speed of information processing.

Conclusions: PCBs, MeHg, and Pb each showed specific and distinct patterns of adverse associations with the outcomes measured during infancy. By contrast, none of these exposures was associated with performance on the BSID-II, a global developmental measure. The more focused, narrow band measures of cognitive function that appeared to be sensitive to these exposures also provide early indications of long-term impairment in specific domains that would otherwise not likely be evident until school age.

Citation: Boucher O, Muckle G, Jacobson JL, Carter RC, Kaplan-Estrin M, Ayotte P, Dewailly É, Jacobson SW. 2014. Domain-specific effects of prenatal exposure to PCBs, mercury, and lead on infant cognition: results from the Environmental Contaminants and Child Development Study in Nunavik. Environ Health Perspect 122:310–316; http://dx.doi.org/10.1289/ehp.1206323

## Introduction

Polychlorinated biphenyls (PCBs), methylmercury (MeHg), and lead (Pb) are ubiquitous environmental contaminants, to which exposure *in utero* has been linked to adverse effects on cognitive function in childhood (Grandjean et al. 1997; Jacobson and Jacobson 1996; Needleman et al. 1979). PCBs are persistent organochlorine industrial compounds that were banned in the 1970s and 1980s. Mercury (Hg) is an element that enters the food chain from both natural and anthropogenic sources and is converted to neurotoxic MeHg by aquatic biota. Environmental exposure to Pb, a metal that is used in numerous commercial products, has declined considerably with the conversion to unleaded gasoline but continues to be associated with poorer intellectual function even at low levels (Canfield et al. 2003; Lanphear et al. 2000).

As a growing number of environmental contaminants are recognized as teratogenic, a key issue is whether such exposures are associated with a global, undifferentiated pattern of cognitive impairment or whether exposure to each substance leads to a distinct profile of deficits based on its particular properties and mechanisms of action. Most studies of Pb have focused on postnatal exposure, primarily during the toddler period, and there is extensive evidence of long-term effects from postnatal Pb exposure on childhood IQ (e.g., Bellinger et al. 1992; Dietrich et al. 1993) and attention (e.g., Chiodo et al. 2004; Plusquellec et al. 2010). In childhood, prenatal PCB exposure has been linked primarily to reduced IQ and reading achievement (Jacobson and Jacobson 1996; Stewart et al. 2008), with some evidence suggesting vulnerability in response inhibition (Stewart et al. 2005). Effects of prenatal MeHg exposure in childhood have been examined in two large prospective studies—one in the Faroe Islands, where Hg was associated with poorer cognitive performance at 7 and 14 years of age (Debes et al. 2006; Grandjean et al. 1997); and the other, in the Seychelles Islands, where few adverse associations were found (Davidson et al. 2011; Myers et al. 1995, 2003). In the Faroese study, exposures were associated with diverse domains, including attention, fine motor function, visual spatial abilities, and memory. Consistent with these findings, we have recently reported an association between higher prenatal MeHg exposure and alterations in auditory attention and visual processing using neurophysiological assessments in school-age Inuit children (Boucher et al. 2010; Ethier et al. 2012). Given confounding between PCB and MeHg exposure, the effects of these two contaminants can be difficult to discriminate from each other (Grandjean et al. 2001).

Relatively few studies have examined effects of prenatal exposure to these contaminants on infant development. Among these contaminants, only PCBs have been consistently linked to a specific domain of infant cognitive function in multiple studies. In a cohort of infants exposed *in utero* to maternal consumption of PCB-contaminated fish, cord blood PCB concentrations were associated with poorer visual recognition memory on the Fagan Test of Infant Intelligence (FTII) at 7 months postpartum (Jacobson et al. 1985). This finding has been replicated at low levels of exposure in Oswego, New York (Darvill et al. 2000), and in infants whose mothers were exposed to high levels of exposure during an industrial accident in Taiwan (Ko et al. 1994). Two studies that examined the effects of prenatal exposure to MeHg in infancy linked that exposure to poorer psychomotor function on the Bayley Scales of Infant Development (BSID) at 30 and 36 months, respectively (Davidson et al. 2008; Lederman et al. 2008). Most of the evidence of cognitive impairment associated with prenatal Pb exposure comes from global developmental assessments, particularly the BSID (Bellinger et al. 1987; Dietrich et al. 1987; Hu et al. 2006), although one study has provided evidence for effects on sustained attention (Plusquellec et al. 2007). The two studies that administered the FTII to Pb-exposed infants yielded contradictory findings (Darvill et al. 2000; Jedrychowski et al. 2008).

The assessment of the adverse effects of contaminants in fish-eating populations is complicated by the fact that contaminated marine foods are also often rich in beneficial nutrients, such as omega-3 polyunsaturated fatty acids (n-3 PUFAs) and selenium (Se). We have previously reported evidence of beneficial effects on development attributable to prenatal n-3 PUFA intake in this cohort (Boucher et al. 2011; Jacobson et al. 2008), which could obscure adverse effects of seafood contaminants. Se may mitigate the effects of Hg (Ganther et al. 1972; Jacobson 1992; Watanabe et al. 1999). Evidence from three studies suggests that statistical adjustment for prenatal intake of these nutrients can reveal associations with contaminants that are not apparent or are weaker than those evident when no statistical adjustment is made (Budtz-Jørgensen et al. 2007; Lederman et al. 2008; Strain et al. 2008).

Jacobson (1998) compared associations of prenatal exposure to PCBs, alcohol, and cocaine with the FTII and found that different patterns of associations with each of these substances could already be detected in infancy. PCB exposure was associated with poorer recognition memory, alcohol with slower cognitive processing speed, and cocaine with faster processing speed but poorer recognition memory. Although specific mechanisms that would explain these patterns of adverse associations remain unknown, these data suggest that administration of narrow band measures of infant cognition in populations exposed to multiple neurotoxicants has the potential to identify specific domains that characterize each exposure and suggest sequelae that might be anticipated at school age.

In the present study we administered two narrow band tests to a sample of Inuit infants in Nunavik (Arctic Québec). This population is among the most highly exposed to PCBs and MeHg on earth because of long-range transport of these compounds via atmospheric and ocean currents and their bioaccumulation in fish and sea mammals that are staples of the Inuit diet (Muckle et al. 2001). Inuit children from Nunavik also have higher blood Pb levels than their southern Canadian counterparts, which has been attributed to the use of Pb pellets for hunting small game (Lévesque et al. 2003). We hypothesized that each contaminant would be associated with a distinct profile of cognitive impairment on these narrow band measures that differs from that associated with the other contaminants. We also hypothesized that the associations of PCBs and MeHg, two seafood contaminants, with the infant outcomes would be stronger after statistical control for prenatal seafood nutrients, particularly n-3 PUFAs.

## Methods

*Participants*. The sample consisted of 94 Inuit infants and their mothers from the Nunavik Environmental Contaminants and Child Development Study. Between November 1995 and March 2001, pregnant women from the three largest Inuit villages on the Hudson Bay coast were invited to participate. Detailed informed consent was obtained from the infant’s mother before participation, following procedures approved by the institutional review boards at Wayne State and Laval Universities, and the study was endorsed by the Nunavik Nutrition and Health Committee and Municipal Councils of the three villages. All infant examiners were blinded to prenatal exposure history. Demographic background, smoking, alcohol and drug use during pregnancy, and other maternal characteristics were ascertained in maternal interviews at mid-pregnancy and 1 month postpartum.

*Infant cognitive assessment*. FTII. The FTII (Fagan and Singer 1983) was administered at 6.5 and 11 months. The infant is shown two identical photos and is then shown the familiar photo paired with a novel one; there are 10 problem sets. Two measures are computed: *a*) Novelty preference, defined as proportion of looking time devoted to the novel stimulus, provides a measure of recognition memory; *b*) fixation duration, defined as the average duration of the infant’s visual fixations to the stimuli, reflects speed of information processing (Colombo et al. 1991; Jacobson SW et al. 1992). Both FTII measures have been shown to be moderately predictive of childhood IQ (McCall and Carriger 1993; McGrath et al. 2004).

A-not-B. In the A-not-B task (Diamond 1990), administered at 11 months, a small toy is placed in one of two embedded wells, which are then hidden under cloth covers. After a delay of 3–11 sec, the infant can retrieve the toy. As the infant grows older, s/he is able to correctly retrieve the toy over progressively longer time delays. The principal outcome is “length of delay–3 correct,” the longest delay at which the child retrieves the toy correctly three times in a row. We also examined “length of delay–2 correct,” correct retrieval twice in a row, and percent perseverative errors on A-not-B trials. A-not-B, which depends on working memory and is believed to reflect early development of executive function, involves the ability to briefly maintain information in memory and to override a prepotent response—in this case, to use visual input to override the impulse to search where the toy was previously found (Espy et al. 1999). Some have argued, however, that A-not-B assesses visually guided reaching (Smith et al. 1999).

Bayley Scales of Infant Development–2nd Edition. The BSID–2nd Edition (BSID-II) (Bayley 1993) is a standardized test, the most widely used global assessment of infant development, and consists of the mental (MDI) and psychomotor (PDI) development indices. The BSID-II was administered at 11 months.

11-year assessments. IQ was assessed on a subset of children (*n* = 64) on the Wechsler Intelligence Scales for Children, 4th Edition (WISC-IV) (Wechsler 2003). The test battery was administered in Inuktitut, the children’s native language, and the scales were adapted for Inuit culture. Because the WISC-IV Verbal Comprehension subtests are not culturally appropriate for the Inuit, verbal proficiency was assessed on two other verbal tests: the Boston Naming Test (Kaplan et al. 1983) and the Delis-Kaplan Verbal Fluency Test (Delis et al. 2001). IQ was estimated based on seven nonverbal WISC-IV subtests and these two more culturally appropriate verbal tests. The validity of the estimated IQ scores was evaluated in a sample of 30 children from metropolitan Detroit, Michigan [15 boys, 15 girls; mean (± SD) age, 10.6 ± 1.4 years], who were administered the standard versions of the WISC-IV, Boston Naming Test, and Delis-Kaplan Verbal Fluency Test, as well as the Inuit version of the Boston Naming Test. The children’s scores on the standard and Inuit versions of the Boston Naming Test were highly correlated, intraclass correlation (ICC) = 0.93. Estimated IQ, when calculated as it had been for the Inuit children, was highly correlated with Full-Scale IQ on the standard version of the WISC-IV, ICC = 0.92. To indicate which children would likely meet DSM-IV (*Diagnostic and Statistical Manual of Mental Disorders, 4th edition*; American Psychiatric Association 2000) criteria for attention deficit/hyperactivity disorder (ADHD), teacher ratings were obtained using the Disruptive Behavior Disorders Scale (*n* = 60) (Pelham et al. 1992).

*Biological samples*. A 30-mL blood sample was obtained from the umbilical cord at delivery. A 10-mL milk sample was collected from breastfeeding mothers at the postnatal interview. PCB-153 (the most prevalent PCB congener) and docosahexaenoic acid (DHA; the n-3 PUFA most strongly implicated in brain development and function) were measured in the cord plasma samples; and Hg, Pb, and Se were measured in cord blood. Contaminant and Se analyses were performed at the Centre de Toxicologie du Québec (Québec City, Québec, Canada) which is accredited by the Canadian Association for Environmental Analytical Laboratories. Detailed analytical procedures are described elsewhere (Muckle et al. 2001) and summarized in the Supplemental Material, p. 2.

*Confounding variables*. The following control variables were assessed for consideration as potential confounders of effects of contaminants on infant development: infant sex and age at assessment; birth weight; maternal age at delivery; parity; social environment [composite measure computed by calculating the average of the *z*-scores obtained for socioeconomic status (SES) (Hollingshead 2011), maternal education (years), and nonverbal reasoning ability (Raven et al. 1992), the Home Observation for Measurement of the Environment (HOME) (Bradley and Caldwell 1979), and language used at interview (Inuktitut/English or French)]; maternal binge drinking (≥ 5 drinks/occasion; yes/no), smoking (cigarettes/day), and frequent marijuana use (4 times/month; yes/no) during pregnancy; and cord DHA and Se concentrations. A composite social environment score was used because these variables were intercorrelated and together contribute synergistically to the quality of the intellectual environment in the home for promoting cognitive development [e.g., adjusted *R*^2^ (coefficient of determination) in a regression model for the prediction of MDI performance was larger for the composite variable entered alone (0.078) than for the sum of each five variables (0.056)]. Each of the three contaminants was also assessed as a potential confounder in all the analyses of the other contaminants.

*Statistical analyses*. The normality of each variable’s distribution was inspected visually and checked for skewness and kurtosis (normality range, –2.0 to 2.0). Natural log transformations were conducted on PCB-153, Hg, Pb, and Se concentrations and parity because they followed log-normal distributions. The following variables with extreme values (> 3 SDs from the mean) were recoded to one point above the highest observed non-outlying value (number of outliers indicated in parentheses): infant age at 11-month assessment (*n* = 4), and cord plasma DHA (*n* = 1; Winer 1971).

We used repeated-measures multiple regression analyses (proc mixed, SAS version 9.2; SAS Institute Inc., Cary, NC) to examine the associations of each of the cord contaminant measures with each of the FTII outcomes (novelty preference and fixation duration). This method adjusts for within-subject variability and allows for pairwise deletion, so that in cases with a missing outcome observation (i.e., either 6.5- or 11-month assessment) the value from the other assessment is included. The associations between the cord contaminant concentrations and the A-not-B and BSID-II outcomes were estimated using hierarchical multiple regression analyses. Age at testing (except for the BSID-II variables which were age-adjusted) and social environment were treated as mandatory covariates in each multivariate model. Additional covariates were included in the models for a given outcome if correlated with that outcome at *p* < 0.10.

To examine confounding by DHA, which may bias associations with contaminants toward the null hypothesis, we adjusted for DHA by entering it at the last step of each analysis. Given the possible protective role of DHA and Se, we reran the multivariate models, adding interaction terms for each of the three contaminants by DHA and for Hg by Se on outcomes found to be affected by the contaminants.

## Results

*Sample characteristics*. Sample characteristics are summarized in Table 1. Approximately one mother in four was < 20 years of age at prenatal interview, and one in five had completed secondary school. A large proportion of mothers smoked tobacco while pregnant. Prenatal exposures to PCB-153, Hg, DHA, and Se were moderately intercorrelated (Pearson correlation coefficients ranged from 0.25 to 0.53, all *p*-values < 0.05), presumably because seafood is a primary source. Pb was not correlated with DHA or Se (all *p*-values > 0.20), presumably because the primary source of Pb exposure is lead pellets lodged in the muscle of small game, rather than seafood.

**Table 1:** Descriptive data for the study sample (*n* = 94).

Variable	*n*	Mean ± SD	Median	Range	Percent
Infant characteristics
Sex (% males)	94				63.8
Age at 6.5-month testing (weeks)	91	30.4 ± 3.5	29.9	25.6–40.9
Age at 11-month testing (weeks)	90	50.5 ± 6.9	47.8	43.2–81.1
Birth weight (g)	94	3566.7 ± 461.8	3,550	2,440–4,560
Gestation duration (weeks)	94	39.2 ± 1.5	39.0	36.0–42.0
Adoption status (% adopted)	94				10.6
Maternal and family characteristics
Age at prenatal interview (years)	94	24.6 ± 5.8	24.3	15.3–39.0
Parity (no. of live births)	94	2.1 ± 1.8	2	0–9
Marital status (% married or living with someone)	94				69.1
Breastfeeding (weeks) (% yes)^*a*^	93	26.3 ± 17.1	24.3	0.1–57.3	87.1
Social environment composite measure^*b*^	94	0.0 ± 0.5	0.1	–1.3–1.3
Maternal education (years)	94	8.9 ± 1.7	9.0	5.5–14.3
Maternal nonverbal reasoning abilities^*c*^	94	34.6 ± 8.1	36	13–48
Socioeconomic status^*d*^	94	25.5 ± 9.5	24.8	8–44
HOME^*e*^	85	31.6 ± 5.2	32	19–41
Language at interview (% Inuktitut)	94				18.1
Maternal consumption during pregnancy
Cigarettes (no./day) (% yes)^*f*^	94	10.8 ± 5.7	10.3	1–25	93.6
Binge drinking (≥ 5 standard drinks of alcohol per occasion) (% yes)	94				39.4
Marijuana (% yes)	94				40.4
Contaminants and nutrients
Cord plasma PCB-153 (μg/kg lipids)	92	114.8 ± 96.0	78.1	12.0–550.9
Cord blood Hg (μg/L)	91	22.5 ± 16.6	17.0	2.4–97.3
Cord blood Pb (μg/dL)	93	4.8 ± 3.5	3.5	0.5–17.8
Cord plasma DHA (% phospholipids)	90	3.7 ± 1.2	3.7	1.3–7.5
Cord blood Se (μg/L)	91	296.4 ± 122.8	268.5	67.9–915.9
FTII
Novelty preference, 6.5 months (%)	89	57.9 ± 6.2	58.0	39.0–74.0
Novelty preference, 11 months (%)	89	59.5 ± 6.3	59.4	38.0–74.0
Fixation duration, 6.5 months (sec)	89	1.9 ± 0.4	1.8	1.0–3.2
Fixation duration, 11 months (sec)	89	1.7 ± 0.3	1.6	1.2–2.6
A-not-B
Length of delay–2 correct	77	1.8 ± 2.2	0	0–9
Length of delay–3 correct	77	1.4 ± 2.0	0	0–7
Perseverative errors (%)	73	19.6 ± 15.6	17.7	0–69
BSID-II
MDI	87	94.2 ± 7.6	95	76–115
PDI	87	89.5 ± 11.5	89	58–120
WISC-IV at 11 years
Full-Scale IQ	64	88.8 ± 8.7	88	72–108
Disruptive Behavior Scale at 11 years
ADHD	60				30.0
^***a***^For breastfed children, at 11-month visit. ^***b***^Computed by calculating the average of the *z*-scores obtained for SES, maternal education, Raven, HOME, and language used at interview. ^***c***^Assessed with the Hollingshead index (Hollingshead 2011). ^***d***^Based on the Raven Progressive Matrices (Raven et al. 1992). ^***e***^Assessed with the HOME inventory (Bradley and Caldwell 1979). ^***f***^For mothers who smoked.					

Pearson correlations between control variables and infant outcomes are presented in Table 2. Age at testing and social environment correlated with several of the outcomes, supporting the decision to systematically control for these variables in the regression models. The predictive validity of the infant measures for child cognitive function and behavior was also examined by modeling the associations with IQ and teacher-rated ADHD at 11 years (as independent variables) with and without adjustment for contaminant and nutrient exposures measured at 11 years of age (see Supplemental Material, Table S1). Shorter FTII fixations at 11 months were related to better performance on the WISC-IV Working Memory Index (standardized β-coefficient = –0.35; 95% CI: –0.62, –0.08) and to Full-Scale IQ (β-coefficient = –0.30; 95% CI: –0.58, –0.03), after adjustment for contaminants and nutrients at 11 years. Higher MDI scores were also associated with higher Full-Scale IQ (β-coefficient = 0.28; 95% CI: 0.01, 0.54).

**Table 2:** Pearson correlations relating confounding variables to infant outcomes (*n* = 94).

Variable	FTII, 6.5 months		FTII, 11 months		A-not-B			BSID-II	
	Novelty preference	Fixation duration	Novelty preference	Fixation duration	Length of delay–2 correct	Length of delay–3 correct	Perseverative errors (%)	MDI	PDI
Infant characteristics
Sex (females = 1; males = 2)	0.05	0.01	–0.02	–0.02	0.05	0.03	0.10	0.04	–0.05
Age at testing	–0.10	–0.14	–0.31**	–0.26*	0.28*	0.27*	–0.32**	0.14	–0.01
Birth weight	0.12	–0.09	–0.05	–0.09	–0.07	–0.01	–0.05	0.19^#^	0.18^#^
Maternal and family characteristics
Age	–0.13	0.12	–0.12	0.12	0.01	0.04	0.19	–0.03	–0.04
Parity	–0.04	–0.05	–0.10	0.08	0.05	0.05	0.03	–0.05	0.09
Social environment	–0.04	–0.38**	0.00	–0.23*	0.14	0.10	–0.15	0.29**	0.15
Maternal consumption during pregnancy
Cigarettes	–0.10	0.01	–0.07	0.16	0.14	0.10	–0.14	0.04	0.13
Binge drinking (0 = no; 1 = yes)	0.05	–0.01	0.11	0.09	0.11	0.13	0.02	0.09	–0.09
Marijuana (0 = no; 1 = yes)	0.05	0.10	–0.09	0.05	0.04	0.00	–0.01	–0.03	–0.25*
Nutrients
Cord plasma DHA (% phospholipids)	0.20^#^	–0.09	–0.03	–0.04	–0.10	–0.10	–0.13	0.19^#^	0.12
Cord blood Se (μg/L)	0.09	0.13	–0.02	0.09	–0.06	–0.05	–0.21^#^	0.10	–0.10
^#^*p* ≤ 0.10. *p ≤ 0.05. **p ≤ 0.01.									

*Associations between contaminants and infant performance*. Table 3 summarizes the results from the regression analyses examining the associations between contaminant exposure and infant performance. PCB-153 was associated with poorer recognition memory on the FTII, as indicated by lower novelty preference. This association reached statistical significance only after control for cord DHA, suggesting that confounding by DHA biased the estimated association toward the null hypothesis. Because PCBs are lipophilic and readily transmitted via breastfeeding, we performed a regression analysis on FTII novelty preference based on breast milk PCB-153 concentrations, breastfeeding duration, and their interaction (Jacobson et al. 1984). Neither breastfeeding duration nor its interaction with PCBs was predictive of this outcome (*p* > 0.20), suggesting that the observed association was attributable primarily to PCB exposure *in utero*.

**Table 3:** Associations between contaminant concentrations in cord blood (ln-transformed) and test performance [β (95% CI)].

Outcomes	PCB-153			Mercury			Lead		
	Model 1	Model 2	Model 3	Model 1	Model 2	Model 3	Model 1	Model 2	Model 3
FTII
Novelty preference	–0.14 (–0.30, 0.02)^#^	–0.14 (–0.30, 0.02)^#^	–0.17 (–0.34, 0.00)*	0.03 (–0.14, 0.20)	0.03 (–0.15, 0.20)	0.00 (–0.19, 0.19)	–0.12 (–0.28, 0.04)	–0.14 (–0.31, 0.03)^#^	–0.13 (–0.31, 0.05)
Fixation duration	0.07 (–0.07, 0.21)	0.09 (–0.05, 0.22)	0.09 (–0.05, 0.24)	0.14 (–0.01, 0.29)^#^	0.11 (–0.03, 0.26)	0.13 (–0.03, 0.29)^#^	0.21 (0.07, 0.35)**	0.15 (0.01, 0.29)*	0.21 (0.07, 0.35)**
A-not-B
Length of delay–2 correct	–0.12 (–0.34, 0.11)	–0.20 (–0.41, 0.02)^#^	–0.15 (–0.36, 0.07)	–0.22 (–0.43, 0.01)^#^	–0.30 (–0.49, –0.07)**	–0.25 (–0.46, 0.00)*	–0.12 (–0.35, 0.11)	–0.11 (–0.35, 0.13)	–0.08 (–0.30, 0.15)
Length of delay–3 correct	–0.14 (–0.36, 0.10)	–0.23 (–0.44, –0.01)*	–0.18 (–0.40, 0.05)	–0.20 (–0.42, 0.03)^#^	–0.28 (–0.49, –0.05)*	–0.22 (–0.45, 0.03)^#^	–0.08 (–0.31, 0.15)	–0.08 (–0.32, 0.16)	–0.05 (–0.28, 0.19)
Perseverative errors (%)	0.10 (–0.14, 0.35)	0.21 (–0.03, 0.47)^#^	0.20 (–0.04, 0.47)^#^	–0.27 (–0.52,–0.04)*	–0.15 (–0.45, 0.13)	–0.21 (–0.53, 0.09)	0.07 (–0.17, 0.31)	0.04 (–0.20, 0.29)	–0.00 (–0.27, 0.27)
BSID-II
MDI	0.08 (–0.15, 0.31)	0.07 (–0.14, 0.29)	0.03 (–0.20, 0.26)	0.10 (–0.13, 0.33)	0.12 (–0.10, 0.35)	0.08 (–0.15, 0.33)	–0.05 (–0.28, 0.17)	0.03 (–0.19, 0.25)	–0.02 (–0.26, 0.23)
PDI	0.00 (–0.22, 0.23)	–0.02 (–0.24, 0.20)	–0.05 (–0.28, 0.18)	0.04 (–0.19, 0.28)	0.04 (–0.19, 0.27)	0.01 (–0.24, 0.25)	–0.09 (–0.32, 0.13)	–0.03 (–0.26, 0.19)	–0.05 (–0.30, 0.20)
Values are standardized regression coefficients (95% CIs) for multiple regression analyses (with repeated measures for FTII measures). Model 1: univariate model; model 2 = multivariable model adjusting for potential confounders (FTII outcomes and A-not-B Length of delay: infant age at testing, social environment composite; A-not-B Perseverative errors: infant age at testing, social environment composite, cord Se; MDI: social environment composite, birth weight; PDI: social environment composite, maternal marijuana during pregnancy); model 3, multivariable model adjusting for potential confounders and DHA. ^#^*p* ≤ 0.10. *p ≤ 0.05. **p ≤ 0.01.									

**Figure 1:**
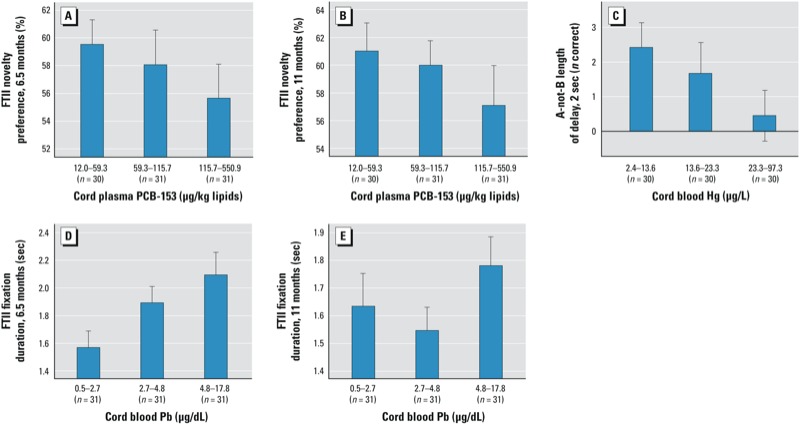
Dose–response analyses relating prenatal PCB exposure to FTII novelty preference at 6.5 (*A*) and 11 (*B*) months, adjusted for child age, social environment, and DHA; (*C*) prenatal Hg to A-not-B Length of delay–2 correct adjusted for child age and social environment; and Pb to FTII fixation duration at 6.5 (*D*) and 11 (*E*) months, adjusted for child age and social environment. Adjusted means were computed by summing the residuals from the each of the regression analyses (including each confounder). Error bars represent ± 1 SE.

After control for confounders, cord blood Hg concentrations were associated with poorer performance on A-not-B (length of delay–2 correct). The association between Hg and length of delay–3 correct fell just short of statistical significance after control for DHA. Prenatal Pb was associated with longer fixation duration on the FTII, suggesting slower information processing speed. None of the contaminant variables was associated with the BSID-II measures. Figure 1 shows mean performance according to tertile of exposure for each significant association between the contaminants and infant outcomes. Except for cord blood Pb and FTII fixation duration at 11 months, associations increased or decreased monotonically with increasing tertiles of exposure

None of the interaction terms for the contaminants with DHA, or Hg with Se, were significant (all *p*-values > 0.10), and there was no pattern of results suggesting antagonistic effects that could not be detected due to low statistical power (data not shown).

## Discussion

In the present study we estimated effects of prenatal exposure to PCBs, Hg, and Pb on cognitive function at 6.5 and 11 months of age in a single cohort. Each contaminant was adversely associated with a different outcome, suggesting neurotoxic impairments that may be mediated by distinct mechanisms. PCB exposure was associated with poorer visual recognition memory; Hg, with poorer A-not-B performance; and Pb, with slower information processing speed. By contrast, these contaminants were not associated with the more global BSID-II MDI and PDI assessments. This specificity is consistent with our previous findings suggesting that the recognition memory deficit is specific to prenatal PCB exposure and different from effects of other neurotoxicants, including alcohol and cocaine (Jacobson 1998).

Our findings are consistent with previous reports based on study populations in Michigan; Taiwan; and Oswego, New York, that prenatal PCB exposure is related to impaired recognition memory on the FTII (Darvill et al. 2000; Jacobson et al. 1985; Ko et al. 1994), and extends this finding to a fourth, ethnically and culturally very different population—the Inuit. PCB levels in this cohort lie between those reported for the Lake Michigan and Oswego studies (Longnecker et al. 2003). Confounding of PCBs and MeHg can make it difficult to differentiate between the effects of these contaminants (Grandjean et al. 2001). In our study, Hg exposure, which was similar to levels reported in the Faroe and Seychelles cohorts (Grandjean et al. 1999; Myers et al. 1995), was not related to recognition memory but rather to A-not-B, which depends on working memory and is an early precursor of executive function. Thus, our findings suggest impairment on distinctively different narrow band end points assessed during infancy. Two previous prenatal Pb studies using the FTII focused only on novelty preference and yielded inconsistent findings (Darvill et al. 2000; Jedrychowski et al. 2008), and Pb exposure was not significantly related to novelty preference in our data. To our knowledge, this is the first study to examine prenatal Pb in relation to FTII processing speed (fixation duration) and to find an association of prenatal Pb with this end point in a group of children with a range of exposures that was relatively low. Like prenatal Pb exposure, fetal alcohol exposure has been associated with slower processing speed (Jacobson et al. 1993; Kable and Coles 2004), which may reflect impaired myelination during early development.

Associations between prenatal PCB exposure and FTII novelty preference in three different cultures—the United States, Taiwan, and Nunavik—suggest a reliable and specific effect on learning and memory that can already be detected in young infants. Prenatal PCB exposure has also been associated with increased short-term memory errors in 4-year-old children (Jacobson JL et al. 1992) and learning and memory deficits in adult rats (Boix et al. 2010; Curran et al. 2011). Experiments on cell cultures have demonstrated perinatal PCB-induced permanent alterations of long-term potentiation in hippocampal cells of rats, which might account for the learning deficits observed in PCB-exposed children and animals (Carpenter et al. 2002; Gilbert 2003; Niemi et al. 1998).

This study is the first to report evidence of adverse effects of prenatal Hg exposure on the A-not-B task at 11 months. Prenatal Hg has been linked to poorer psychomotor development at 30 and 36 months, but not at 9, 12, or 24 months and was not related to A-not-B at 25 months (Davidson et al. 2008; Lederman et al. 2008). Certain time windows may be more sensitive for detecting Hg-related neurotoxic effects on particular tests or functional domains, which may explain some of the inconsistencies observed across studies.

This study also provides further empirical evidence that statistical control for seafood nutrients can improve estimation of the adverse effects of prenatal exposure to seafood contaminants. Failure to control for DHA would have prevented us from detecting significant associations between PCBs and FTII novelty preference. Statistical control for seafood nutrients also led to detection of stronger adverse associations between prenatal Hg exposure and cognitive function in previous studies. In a recent Seychelles study, the association between Hg and 30-month psychomotor function was not significant until adjustment was made for n-3 PUFAs (Strain et al. 2008); in a lower Manhattan study, associations with 36-month PDI and 48-month IQ were not evident until maternal seafood consumption during pregnancy was adjusted (Lederman et al. 2008); and in the Faroes, control for maternal fish consumption during pregnancy strengthened adverse associations between prenatal MeHg exposure and cognitive performance at 7 and 14 years (Budtz-Jørgensen et al. 2007). This converging evidence underscores the importance of measuring seafood nutrients when evaluating neurotoxic effects of exposure to seafood contaminants (Valera et al. 2009).

Although our findings suggest specific patterns of adverse effects resulting from prenatal PCBs, MeHg, and Pb exposures, some limitations need to be considered. The study was conducted on a relatively small sample of children, thereby limiting statistical power. Our infant assessment was limited to three tests; a more comprehensive assessment might have revealed additional affected domains or some overlapping effects of these contaminants. Our study suggests that each neurotoxic contaminant may have a distinct “behavioral signature,” but situational, subject-related, and environmental factors can influence a chemical’s toxicity. Because cohort studies often differ on these factors, specific outcomes affected by a given exposure may vary from one study to another, which may explain some of the inconsistencies found across studies (Bellinger 1994). We were able to control for an extensive list of potential confounders, such as maternal alcohol, drug, and cigarette use during pregnancy, but lacked measures of postnatal exposure to MeHg and Pb because they were not assessed at 6 months. We also did not assess iron status at birth or during infancy. Because iron contributes to brain and cognitive development (Carter et al. 2010; Lozoff 2007) and iron deficiency may increase Pb absorption (Rahman et al. 2012), iron status should be assessed in future studies.

## Conclusions

In this study we examined effects of prenatal exposure to three neurotoxicants on infant cognitive development using neurobehavioral tasks assessing different domains of cognition. Each contaminant was independently associated with impairment of distinct aspects of cognitive function with long-term implications for cognitive development—PCBs with visual recognition memory, MeHg with working memory and an early precursor of executive function, Pb with processing speed—deficits that can already be detected during the first year of life. These data provide compelling evidence for the utility of narrow band measures of infant cognition in studies of neurotoxic pollutants. Early detection of adverse effects can permit faster recognition of a substance’s neurotoxicity, thereby promoting more rapid interventions aimed at reducing exposure in populations at risk. These findings also underscore the importance of adjusting for co-exposure to seafood nutrients, particularly DHA, when estimating effects of exposure to contaminants in fish and sea mammals to avoid the risk of failing to detect adverse effect on public health. Finally, our study supports current public health recommendations in Nunavik aimed at limiting consumption of highly contaminated traditional foods (e.g., marine mammal fat and meat), especially among women of childbearing age. Because of their low contaminant levels and high fatty acids concentrations, finfish species, such as Arctic char, which are also part of the traditional Inuit diet, would constitute good substitutes for these foods.

## Supplemental Material

(201 KB) PDF

## References

[r1] American Psychiatric Association. (2000). Diagnostic and Statistical Manual of Mental Disorders, 4th Edition.

[r2] Bayley N. (1993). Bayley Scales of Infant Development.. San Antonio, TX:Psychological Corporation.

[r3] Bellinger D (1994). Interpreting the literature on lead and child development: the neglected role of the experimental system.. Neurotoxicol Teratol.

[r4] Bellinger D, Leviton A, Waternaux C, Needleman H, Rabinowitz M (1987). Longitudinal analyses of prenatal and postnatal lead exposure and early cognitive development.. N Engl J Med.

[r5] Bellinger DC, Stiles KM, Needleman HL (1992). Low-level lead exposure, intelligence and academic achievement: a long-term follow-up study.. Pediatrics.

[r6] Boix J, Cauli O, Felipo V (2010). Developmental exposure to polychlorinated biphenyls 52, 138 or 180 affects differentially learning or motor coordination in adult rats. Mechanisms involved.. Neuroscience.

[r7] Boucher O, Bastien CH, Saint-Amour D, Dewailly É, Ayotte P, Jacobson JL (2010). Prenatal exposure to methylmercury and PCBs affects distinct stages of information processing: an event-related potentials study with Inuit children.. Neurotoxicology.

[r8] Boucher O, Burden MJ, Muckle G, Saint-Amour D, Ayotte P, Dewailly É (2011). Neurophysiological and neurobehavioral evidence of beneficial effects of prenatal omega-3 fatty acid intake on memory function at school age.. Am J Clin Nutr.

[r9] Bradley RH, Caldwell BM (1979). Home observation for measurement of the environment: a revision of the preschool scale.. Am J Ment Defic.

[r10] Budtz-Jørgensen E Grandjean P Weihe P 2007 Separation of risks and benefits of seafood intake. Environ Health Perspect 115 323 327; 10.1289/ehp.9738 17431478 PMC1849938

[r11] Canfield RL, Henderson CR, Cory-Slechta DA, Cox C, Jusko TA, Lanphear BP (2003). Intellectual impairment in children with blood lead concentrations below 10 μg per deciliter.. N Eng J Med.

[r12] Carpenter DO Hussain RJ Berger DF Lombardo JP Park H-Y 2002 Electrophysiologic and behavioral effects of perinatal and acute exposure to rats to lead and polychlorinated biphenyls. Environ Health Perspect 110 (suppl 3 377 386 12060832 10.1289/ehp.02110s3377 PMC1241186

[r13] Carter RC, Jacobson JL, Burden MJ, Armony-Sivan R, Dodge NC, Angelilli ML (2010). Iron deficiency anemia and cognitive function in infancy.. Pediatrics.

[r14] Chiodo LM, Jacobson SW, Jacobson JL (2004). Neurodevelopmental effects of postnatal lead exposure at very low levels.. Neurotoxicol Teratol.

[r15] Colombo J, Mitchell DW, Coldren JT, Freeseman LJ (1991). Individual differences in infant visual attention: are short lookers faster processors or feature processors?. Child Dev.

[r16] Curran CP Nebert DW Genter MB Patel KV Schaefer TL Skelton MR 2011 *In utero* and lactational exposure to PCBs in mice: adult offspring show altered learning and memory depending on *Cyp1a2* and *Ahr* genotypes. Environ Health Perspect 119 1286 1293; 10.1289/ehp.1002965 21571617 PMC3230394

[r17] Darvill T, Lonky E, Reihman J, Stewart P, Pagano J (2000). Prenatal exposure to PCBs and infant performance on the Fagan Test of Infant Intelligence.. Neurotoxicology.

[r18] Davidson PW, Cory-Slechta DA, Thurston SW, Huang LS, Shamlaye CF, Gunzler D (2011). Fish consumption and prenatal methylmercury exposure: cognitive and behavioral outcomes in the main cohort at 17 years from the Seychelles child development study.. Neurotoxicology.

[r19] Davidson PW, Strain JJ, Myers GJ, Thurston SW, Bonham MP, Shamlaye CF, et al. (2008). Neurodevelopmental effects of maternal nutritional status and exposure to methylmercury from eating fish during pregnancy.. Neurotoxicology.

[r20] Debes F, Budtz-Jørgensen E, Weihe P, White RF, Grandjean P (2006). Impact of prenatal methylmercury exposure on neurobehavioral function at age 14 years.. Neurotoxicol Teratol.

[r21] Delis DC, Kaplan E, Kramer JH. (2001). Delis-Kaplan Executive Function System (D-KEFS).. San Antonio, TX:Psychological Corporation.

[r22] Diamond A (1990). The development and neural bases of memory functions, as indexed by the A-not-B and delayed response tasks, in human infants and infant monkeys.. Ann NY Acad Sci.

[r23] Dietrich KN, Berger OG, Succop PA, Hammond PB, Bornschein RL (1993). The developmental consequences of low to moderate prenatal and postnatal lead exposure: intellectual attainment in the Cincinnati Lead Study Cohort following school entry.. Neurotoxicol Teratol.

[r24] Dietrich KN, Krafft KM, Bornschein RL, Hammond PB, Berger O, Succop PA (1987). Low-level fetal lead exposure effect on neurobehavioral development in early infancy.. Pediatrics.

[r25] Espy KA, Kaufman PM, McDiarmid MD, Glisky ML (1999). Executive functioning in preschool children: performance on A-not-B and other delayed response format tasks.. Brain Cogn.

[r26] Ethier AA, Muckle G, Bastien C, Dewailly É, Ayotte P, Arfken C (2012). Effects of environmental contaminant exposure on visual brain development: a prospective electrophysiological study in school-aged children.. Neurotoxicology.

[r27] Fagan JF, Singer LT. (1983). Infant recognition memory as a measure of intelligence. In: Advances in Infant Research (Lipsitt LP, Rovee-Collier CK, eds).

[r28] Ganther HE Goudie C Sunde ML Kopecky M Wagner P Oh SH 1972 Selenium: relation to decreased toxicity of methylmercury in diets containing tuna. Science 175 1122 5062150 10.1126/science.175.4026.1122

[r29] Gilbert ME (2003). Perinatal exposure to polychlorinated biphenyls alters excitatory synaptic transmission and short-term plasticity in the hippocampus of the adult rat.. Neurotoxicology.

[r30] Grandjean P, Budtz-Jørgensen E, White RF, Jorgensen PJ, Weihe P, Debes F (1999). Methylmercury exposure biomarkers as indicators of neurotoxicity in children aged 7 years.. Am J Epidemiol.

[r31] Grandjean P, Weihe P, Burse VW, Needham LL, Storr-Hansen E, Heinzow B (2001). Neurobehavioral deficits associated with PCB in 7-year-old children prenatally exposed to seafood neurotoxicants.. Neurotoxicol Teratol.

[r32] Grandjean P, Weihe P, White RF, Debes F, Araki S, Yokoyama K (1997). Cognitive deficit in 7-year-old children with prenatal exposure to methylmercury.. Neurotoxicol Teratol.

[r33] Hollingshead AB (2011). Four factor index of social status.. Yale J Sociol.

[r34] Hu H Tellez-Rojo MM Bellinger D Smith D Ettinger AS Lamadrid-Figueroa H 2006 Fetal lead exposure at each stage of pregnancy as a predictor of infant performance. Environ Health Perspect 114 1730 1735; 10.1289/ehp.9067 17107860 PMC1665421

[r35] Jacobson JL, Fein GG, Jacobson SW, Schwartz PM, Dowler JK (1984). The transfer of polychlorinated and polybrominated biphenyls across the human placenta and into maternal milk.. Am J Public Health.

[r36] Jacobson JL, Jacobson SW (1996). Intellectual impairment in children exposed to polychlorinated biphenyls *in utero.*. N Engl J Med.

[r37] Jacobson JL, Jacobson SW, Muckle G, Kaplan-Estrin M, Ayotte P, Dewailly É (2008). Beneficial effects of a polyunsaturated fatty acid on infant development: evidence from the Inuit of Arctic Quebec.. J Pediatr.

[r38] Jacobson JL, Jacobson SW, Padgett RJ, Brumitt GA, Billings RL (1992). Effects of prenatal PCB exposure on cognitive processing efficiency and sustained attention.. Dev Psychol.

[r39] Jacobson K. (1992). Synergistic effects of selenium and mercury on Japanese medaka (*Oryzias latipes*) embryonic development. Abstracts: 43rd International Science and Engineering Fair, Nashville, TN, 10–16 May 1992.

[r40] Jacobson SW (1998). Specificity of neurobehavioral outcomes associated with prenatal alcohol exposure.. Alcohol Clin Exp Res.

[r41] Jacobson SW, Fein GG, Jacobson JL, Schwartz PM, Dowler JK (1985). The effect of intrauterine PCB exposure on visual recognition memory.. Child Dev.

[r42] Jacobson SW, Jacobson JL, O’Neill JM, Padgett RJ, Frankowski JJ, Bihun JT (1992). Visual expectation and dimensions of infant information processing.. Child Dev.

[r43] Jacobson SW, Jacobson JL, Sokol RJ, Martier SS, Ager JW (1993). Prenatal alcohol exposure and infant information processing ability.. Child Dev.

[r44] Jedrychowski W, Perera F, Jankowski J, Rauh V, Flak E, Caldwell KL (2008). Prenatal low-level lead exposure and developmental delay of infants at age 6 months (Krakow inner city study).. Int J Hyg Environ Health.

[r45] Kable JA, Coles CD (2004). The impact of prenatal alcohol exposure on neurophysiological encoding of environmental events at six months.. Alcohol Clin Exp Res.

[r46] Kaplan E, Goodglass H, Weintraub S. (1983). Boston Naming Test.

[r47] Ko HC, Yao B, Chang FM, Hsu CC, Jacobson SW, Jacobson JL. (1994). Preliminary evidence of recognition memory deficits in infant born to Yu-Cheng exposed women. In: Dioxin ’94 (Fiedler H, Huntziger O, Birnbaum L, Lambert G, Needham L, Sax J, eds).

[r48] Lanphear BP, Dietrich K, Auinger P, Cox C (2000). Cognitive deficits associated with blood lead concentrations < 10 μg/dL in US children and adolescents.. Public Health Rep.

[r49] Lederman SA, Jones RL, Caldwell KL, Rauh V, Sheets SE, Tang D, et al. 2008 Relation between cord blood mercury levels and early child development in a World Trade Center cohort. Environ Health Perspect 116 1085 1091; 10.1289/ehp.10831 18709170 PMC2516590

[r50] Lévesque B, Duchesne JF, Gariepy C, Rhainds M, Dumas P, Scheuhammer AM (2003). Monitoring of umbilical cord blood lead levels and sources assessment among the Inuit.. Occup Environ Med.

[r51] Longnecker MP Wolff MS Gladen BC Brock JW Grandjean P Jacobson JL 2003 Comparison of polychlorinated biphenyl levels across studies of human neurodevelopment. Environ Health Perspect 111 65 70; 10.1289/ehp.5463 12515680 PMC1241307

[r52] Lozoff B (2007). Iron deficiency and child development.. Food Nutr Bull.

[r53] McCall RB, Carriger MS (1993). A meta-analysis of infant habituation and recognition memory as predictors of later IQ.. Child Dev.

[r54] McGrath E Wypij D Rappaport LA Newburger JW Bellinger DC 2004 Prediction of IQ and achievement at age 8 years from neurodevelopmental status at age 1 year in children with D-transposition of the great arteries. Pediatrics 114: e572 e576 15492354 10.1542/peds.2003-0983-L

[r55] Muckle G, Ayotte P, Dewailly É, Jacobson SW, Jacobson JL (2001). Prenatal exposure of the northern Québec Inuit infants to environmental contaminants.. Environ Health Perspect.

[r56] Myers GJ, Davidson PW, Cox C, Shamlaye CF, Palumbo D, Cernichiari E (2003). Prenatal methylmercury exposure from ocean fish consumption in the Seychelles child development study.. Lancet.

[r57] Myers GJ, Marsh DO, Davidson PW, Cox C, Shamlaye CF, Tanner MA (1995). Main neurodevelopmental study of Seychellois children following *in utero* exposure to methylmercury from a maternal fish diet: outcome at six months.. Neurotoxicology.

[r58] Needleman HL, Gunnoe C, Leviton A, Reed R, Peresie H, Maher C (1979). Deficits in psychologic and classroom performance of children with elevated dentine lead levels.. N Engl J Med.

[r59] Niemi WD, Audi J, Bush B, Carpenter DO (1998). PCBs reduce long-term potentiation in the CA1 region of rat hippocampus.. Exp Neurol.

[r60] Pelham WE, Gnagy E, Greenslade KE, Milich R (1992). Teacher ratings of DSM-III-R symptoms for the disruptive behavior disorders.. J Am Acad Child Adolesc Psychiatry.

[r61] Plusquellec P, Muckle G, Dewailly É, Ayotte P, Bégin G, Desrosiers C (2010). The relation of environmental contaminants exposure to behavioral indicators in Inuit preschoolers in Arctic Quebec.. Neurotoxicology.

[r62] Plusquellec P, Muckle G, Dewailly É, Ayotte P, Jacobson SW, Jacobson JL (2007). The relation of low-level prenatal lead exposure to behavioral indicators of attention in Inuit infants in Arctic Quebec.. Neurotoxicol Teratol.

[r63] Rahman MA, Rahman B, Ahmad MS, Blann A, Ahmed N (2012). Blood and hair lead in children with different extents of iron deficiency in Karachi.. Environ Res.

[r64] Raven JC, Court JH, Raven J. (1992). Manual for Raven’s Progressive Matrices and Vocabulary Scales: Standard Progressive Matrices.

[r65] Smith LB, Thelen E, Titzer R, McLin D (1999). Knowing in the context of acting: the task dynamics of the A-not-B error.. Psychol Rev.

[r66] Stewart PW Lonky E Reihman J Pagano J Gump BB Darvill T 2008 The relationship between prenatal PCB exposure and intelligence (IQ) in 9-year-old children. Environ Health Perspect 116 1416 1422; 10.1289/ehp.11058 18941588 PMC2569105

[r67] Stewart P, Reihman J, Gump B, Lonky E, Darvill T, Pagano J (2005). Response inhibition at 8 and 9 ½ years of age in children prenatally exposed to PCBs.. Neurotoxicol Teratol.

[r68] Strain JJ, Davidson PW, Bonham MP, Duffy EM, Stokes-Riner A, Thurston SW (2008). Associations of maternal long-chain polyunsaturated fatty acids, methyl mercury, and infant development in the Seychelles Child Development Nutrition Study.. Neurotoxicology.

[r69] Valera B, Dewailly É, Poirier P (2009). Environmental mercury exposure and blood pressure among Nunavik Inuit adults.. Hypertension.

[r70] Watanabe C, Yin K, Kasanuma Y, Satoh H (1999). In utero expose to methylmercury and Se deficiency converge on the neurobehavioral outcome in mice.. Neurotoxicol Teratol.

[r71] Wechsler D. (2003). Wechsler Intelligence Scales for Children. 4th ed.. San Antonio:Psychological Corporation.

[r72] Winer BJ. (1971). Statistical Principles in Experimental Design. 2nd ed.. New York: McGraw-Hill.

