# Supplementary material for: Domain-Specific Effects of Prenatal Exposure to PCBs, Mercury, and Lead on Infant Cognition: Results from the Environmental Contaminants and Child Development Study in Nunavik
Source: Environ Health Perspect. 2014 Jan 17;122(3):310–6. doi: 10.1289/ehp.1206323 (PMC3948023; doi:10.1289/ehp.1206323)
Supplement: (201 KB) PDF [file ehp.1206323.s001.pdf]

Supplemental Material

Domain-Specific Effects of Prenatal Exposure to PCBs, Mercury, and Lead on Infant Cognition: Results from the Environmental Contaminants and Child Development Study in Nunavik

Olivier Boucher, Gina Muckle, Joseph L. Jacobson, R. Colin Carter, Melissa Kaplan-Estrin, Pierre Ayotte, Éric Dewailly, and Sandra W. Jacobson

Table of contents

Analysis of blood and plasma specimens p. 2

Supplemental Material, Table S1 p. 3

References p. 4

Analysis of blood and plasma specimens

PCB congener concentrations were measured in cord plasma and breast milk. The 14 most prevalent PCB congeners (International Union of Pure and Applied Chemistry nos. 28, 52, 99, 101, 105, 118, 128, 138, 153, 156, 170, 180, 183, and 187) were measured in the purified extracts with an HP 5890 high-resolution gas chromatograph equipped with dual-capillary columns (HP Ultra I and Ultra II) and dual Ni-63 electron capture detectors (Hewlett-Packard, Palo Alto, CA, USA). Limits of detection (LODs) were approximately 0.02 μg/L for all compounds. For the current study, PCB 153, which was expressed on a lipid basis, was used as an indicator of total PCB exposure because it is highly correlated with other PCB congeners and is considered an adequate marker of exposure to environmental PCB mixtures in the Arctic (Ayotte et al. 2003; Muckle et al. 2001).

Hg, Pb, and Se concentrations were determined in cord blood. Total Hg concentrations were determined by cold vapor atomic absorption spectrometry (Model 120; Pharmacia, Piscataway, NJ, USA). Pb was determined by graphite furnace atomic absorption with Zeeman background correction (model ZL 4100; Perkin Elmer, Norwalk, CT, USA). LODs were 1.0 nmol/L for Hg, and 50 nmol/L for Pb. Blood Se concentrations were determined with inductively coupled plasma mass spectrometry (ICP-MS) using state-of-the-art instrumentation (PE Elan 6000; Perkin Elmer). The fatty acid composition of cord and maternal plasma phospholipids was determined by the Lipid Analytical Laboratory at the University of Guelph (B.J. Holub), as described by Jacobson and colleagues (2008).

Supplemental Material, Table S1. Associations between infant outcomes and child assessments conducted at 11 years [standardized β-coefficient (95% CI)].

Outcomes	FTII Fixation duration (6.5 months): Unadjusted	FTII Fixation duration (6.5 months): Adjusted	FTII Fixation duration (11 months): Unadjusted	FTII Fixation duration (11 months): Adjusted	MDI: Unadjusted	MDI: Adjusted
Estimated IQ	-0.10 (-0.36, 0.17)	-0.12 (-0.39, 0.16)	-0.24 (-0.49, 0.01)†	-0.30 (-0.58, -0.03) *	0.28 (0.02, 0.53)*	0.28 (0.01, 0.54)*
WISC-IV WMI	-0.01 (-0.28, 0.25)	-0.00 (-0.27, 0.27)	-0.29 (-0.54, -0.04)*	-0.35 (-0.62, -0.08)*	0.23 (-0.03, 0.49)†	0.24 (-0.02, 0.51) †
Digit Span subtest	0.08 (-0.22, 0.40)	0.07 (-0.23, 0.39)	-0.17 (-0.49, 0.11)	-0.25 (0.07, -0.61)†	0.21 (-0.05, 0.55)	0.21 (-0.07, 0.55)
Arithmetic subtest	-0.14 (-0.40, 0.12)	-0.11 (-0.38, 0.16)	-0.33 (-0.58, -0.09) **	-0.34 (-0.60, -0.07)**	0.17 (-0.10, 0.43)	0.20 (-0.07, 0.46)
ADHD diagnosis (based on teacher ratings)	0.23 (-0.04, 0.49)	0.23 (-0.05, 0.51)	0.21 (-0.05, 0.48)	0.25 (-0.04, 0.54) †	-0.17 (-0.44, 0.10)	-0.19 (-0.47, 0.10)

$^{**}p < 0.01$; $^{*}p < 0.05$; $^{\dagger}p < 0.10$.

Values are regression coefficients and 95% confidence intervals for the prediction of 11-year outcomes with infant measures as snutrients include DHA and Se. Natural log transformations were conducted on PCB-153, Hg, and Pb values.

Abbreviations: ADHD, attention deficit hyperactivity disorder; FTII, Fagan Test of Infant Intelligence; MDI, Mental Development Index; WISC-IV WMI, Wechsler Intelligence Scales for Children-IV – Working Memory Index.

References

Ayotte P, Muckle G, Jacobson JL, Jacobson SW, Dewailly E. 2003. Assessment of pre- and postnatal exposure to polychlorinated biphenyls: lessons from the Inuit cohort study. Environ Health Perspect 111:1253-1258.

Delis DC, Kaplan E, Kramer JH. 2001. Delis-Kaplan Executive Function System (D-KEFS). San Antonio, Tx:The Psychological Corporation.

Jacobson JL, Jacobson SW, Muckle G, Kaplan-Estrin M, Ayotte P, Dewailly E. 2008. Beneficial effects of a polyunsaturated fatty acid on infant development: evidence from the Inuit of Arctic Quebec. J Pediatr 152:356-364.

Kaplan E, Goodglass H, Weintraub S. 1983. Boston Naming Test. Philadelphia:Lea & Febiger.

Muckle G, Ayotte P, Dewailly E, Jacobson SW, Jacobson JL. 2001. Prenatal exposure of the northern Québec Inuit infants to environmental contaminants. Environ Health Perspect 109:1291-1299.
